# Syncope in a female patient. Echocardiography and cardiac computed tomography reveal an unexpected diagnosis

**DOI:** 10.1002/ccr3.2164

**Published:** 2019-05-07

**Authors:** Sorin Giusca, Sebastian Buss, Felix Lasitschka, Matthias Karck, Grigorios Korosoglou

**Affiliations:** ^1^ Department of Cardiology & Vascular Medicine GRN Hospital Weinheim Weinheim Germany; ^2^ Radiology Center Heidelberg Heidelberg Germany; ^3^ Institute of Pathology University of Heidelberg Heidelberg Germany; ^4^ Department of Cardiac Surgery University of Heidelberg Heidelberg Germany

**Keywords:** cardiac CT, cardiac imaging, cardiac tumor, embolic risk, fibroelastoma

## Abstract

Papillary fibroelastoma is a benign cardiac tumor with a high potential of embolization. Coronary computed tomography is a valuable tool for the work‐up of patients with papillary fibroelastoma, providing accurate information regarding the coronary circulation and morphology of the cardiac mass.

## 1 INTRODUCTION

Cardiac tumors are increasingly more often diagnosed with currently available imaging techniques. Although still rare entities, they have an important clinical significance because of possible complications such as heart failure, valve dysfunction, and peripheral embolization.^1^, ^2^ Cardiac myxomas were long considered to be the most often encountered benign cardiac tumors.^3^ However, a recent series analysis from the Mayo Clinic challenged these data, as they identified papillary fibroelastomas (PFE) to be the most frequent benign cardiac tumor.^4^ Due to their high embolic risk, PFE should be considered in the presence of cryptogenic stroke.^5^

Transthoracic echocardiography (TTE) is the first‐line diagnostic tool employed for the diagnosis of PFE and cardiac tumors in general. It provides information on the localization, dimension, and mobility of the tumor as well as its effect on the surrounding cardiac structures.^6^ However, transthoracic echocardiography can miss small PFE and the image acquisition is highly dependent on operator experience and patient's echogenic window. Transesophageal echocardiography (TEE) usually provides better image quality and can more precisely delineate the characteristic of PFE. Still, TEE is sometimes less accurate in the evaluation of right heart structures and provides limited information on tissue composition. Cardiac MRI (CMR) and cardiac CT (CCT) usually complement the evaluation of cardiac tumors, including PFE.^7^ They have a higher field of view and can provide information regarding the tumor composition. In this regard, CMR was shown to be an excellent tool for tissue characterization in patients with cardiac tumors.^8^ CCT, on the other hand, can simultaneously evaluate the coronary status of the patient prior to cardiac surgery in low‐ and intermediate‐risk patients.

The surgical excision of the PFE provides a curative treatment and the histological examination of the excised tissue is used to establish the definite diagnosis. A small rate of recurrence has been described in patients with PFE who underwent surgical removal of the tumor.^4^ Herein, we present here the case of a 63‐year‐old patient who was diagnosed with a PFE with a rare localization.

## 2 CASE PRESENTATION

**Figure 1:**
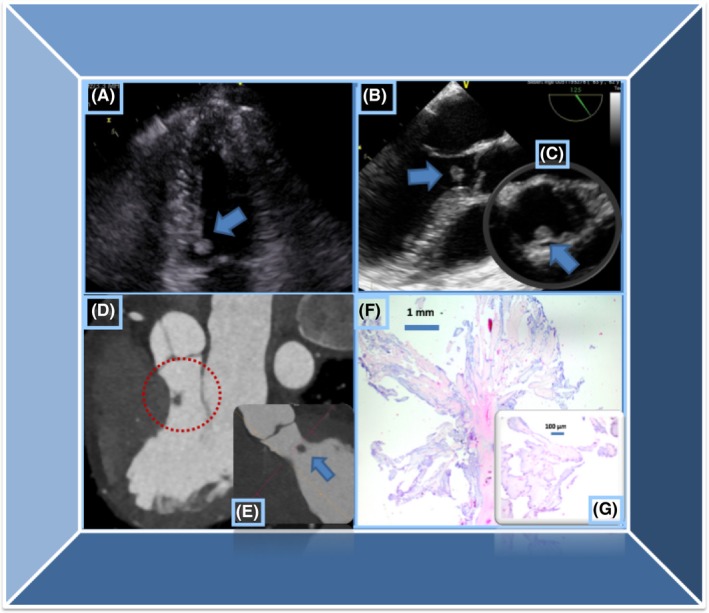
A, Transthoracic echocardiography modified four‐chamber view. Note the structure (blue arrow) in the left ventricular outflow tract. B, Transesophageal echocardiography. Mid‐esophageal view at 120°. Note the same structure present in the LVOT with a probable insertion in the LVOT. C, Transesophageal echocardiography, upper‐esophageal view. Note the prolapse of the cardiac tumor through the aortic valve during systole (white arrow). D, Contrast multidetector CT confirming PFE attachment at the LVOT. F‐E. Histological analysis confirming the diagnosis of PFE. LVOT, left ventricular outflow tract; PFE, papillary fibroelastomas

A 63‐year‐old patent presented in our emergency department after a syncope that occurred immediately after physical exertion, while stepping off an ergometer after a spinning class. The syncope leads to laceration injury at the head of the patient. On presentation, the patient was hemodynamically stable, awake, and oriented. She reported this to be the first event of syncope. The event was not accompanied by angina pectoris, dyspnea, or palpitations. No neurological deficit was noted immediately after she regained consciousness. Furthermore, the patient had no history of cardiovascular disease and no risk factors, whereas her family history was uneventful. Clinical examination, including a complete neurological examination, showed normal findings. Blood pressure, pulse, body temperature, and oxygen saturation in peripheral blood were all within normal range. The ECG showed a normal sinus rhythm without any evidence of ischemic changes, conduction delays, or repolarization abnormalities. The laboratory tests on admission were unspectacular except for a mild elevation of high‐sensitive troponin of 16.9 pg/mL (Reference <14 pg/mL), with the value remaining stable on further testing. Being a high‐risk syncope based on the recently published ESC guidelines, the patient was admitted with continuous monitoring.^9^ On the same day, a TTE was performed showing a hypermobile mass in the left ventricular outflow tract (LVOT), which measured 6 × 6 mm (Figure 1A). The structure was appearing to prolapse through the aortic valve during systole. No aortic stenosis or regurgitation was present. In addition, the tumor was not obstructing the LVOT and no relevant gradient was measured in LVOT at rest. In addition, hypertrophy of the basal septum could not be detected, and systolic anterior motion of the mitral valve leaflet was not present. TEE confirmed the presence of a hypermobile, slightly hyperechogenic structure, which appeared to be attached to the LVOT (Figure 1B). The structure prolapsed through the aortic valve during systole (Figure 1C). Taken together, the suspicion of PFE was raised.^6^ However, several differential diagnoses were considered. Firstly, a ventricular thrombus would have been very unlikely as the patient did not have any history of myocardial infarction and no wall motion abnormalities were identified by echocardiography. A cardiac myxoma, on the other hand, was also considered. Nevertheless, localization of cardiac myxomas in the LVOT would be highly atypical.^10^ Due to the high embolic risk of PFE, the decision was made for surgical removal of the tumor. Due to the low risk for coronary artery disease based on clinical presentation and a possible risk of embolization during invasive angiography, the patient was referred for coronary computed tomography angiography (CCTA). CCTA exhibited normal coronary arteries and clearly showed attachment of the PFE to the LVOT (Figure 1D). In addition, a brain magnetic resonance imaging (MRI) was performed, which excluded recent and older cerebral ischemic events. In addition, duplex sonography of the carotid arteries exhibited no relevant plaques or stenosis of the internal and external carotid or vertebral arteries.

The tumor was removed by open heart surgery without complications. Histology confirmed the presence of a papillary fibroelastoma, exhibiting avascular branching fronds, which consisted of dense fibroelastic tissue surrounded by layers of endothelial cells (Figure 1E‐F). The postoperative course was uneventful. The patient underwent follow‐up echocardiography at 6 months of follow‐up, showing overall normal findings and no tumor recurrence.

## 3 DISCUSSION

The widespread use of echocardiography in the last decades as a main diagnostic tool in cardiology allowed for better identification and characterization of cardiac tumors. Myxomas, PFE, and lipomas are the most frequently diagnosed benign cardiac tumors. Based on previous autopsy data, cardiac myxomas are considered as the most common benign cardiac tumor.^11^ More recent reports, however, revealed a higher prevalence of PFE compared to myxomas.^4^

The clinical presentation varies from asymptomatic incidental findings to catastrophic events, like myocardial infarction with cardiogenic shock and massive stroke. Embolic events are a frequent occurrence of PFE with the cerebral territory being the most affected one.^12^ Thus, PFE should be considered in patients with an unexplained embolic event. As in our patient, many reports reveal syncope as the main clinical symptom with PFE.^13^, ^14^ Although a clear causality between PFE and syncope has not been established so far, the excision of the tumor results in resolution of such symptoms in most patients. In our patient, however, the syncope might be explained through a dynamic obstruction of the LVOT during exercise.^15^

Most of the PFE (>90%) are located on the left side of the heart and more than two thirds are found on cardiac valves, with the aortic valve being the most frequent.^12^ They usually measure around 10 mm and often present as single tumors. However, cases of multiple PFE have also been reported.^6^ The embolic events result by either parts of the tumor or thrombotic debris that forms on the surface of the PFE. Therefore, medication with aspirin may be useful in such patients until surgery is performed.

Echocardiography is the cornerstone of diagnosis for this type of tumor. A combination of TTE and TEE provides sufficient information about the localization, dimension, and relation with other cardiac structures. In addition, CMR and cardiac CT can prove useful in establishing a definite diagnosis, especially in case of inconclusive findings. Thus, CMR was shown to be a valuable tool in the evaluation of patients with cardiac tumors.^16^ Cardiac CT, on the other hand, proved useful for the evaluation of our patient. It provided an excellent tomographic view of the tumor with a clear identification of the insertion point of the PFE. Several recent reports noted the usefulness of cardiac CT for providing more in‐depth information related to the type of tissue involved.^10^, ^17^ In addition, for the perioperative assessment of patients planned for tumor excision, it can provide a useful alternative to invasive coronary diagnosis. This is especially important in cases where the tumor is located close to the aortic valve apparatus, bearing the risk of an embolic vent during cardiac catheterization.^18^

Several differential diagnoses need to be considered when establishing the therapeutic approach in patients with suspected PFE. Lambl's excrescences are small mobile structures attached to cardiac valves. They are often seen in TEE, and they differentiate from PFE through their “strand‐like” appearance and their localization mostly on the line of closure of the cardiac valve.^19^ A cardiac myxoma could also have the same appearance as a PFE. However, the majority of myxomas are found in the left atrium and are inserted in the interatrial septum. Moreover, the tissue characterization as provided by CMR can help to differentiate between these tumors.^8^, ^20^ Lastly, cardiac thrombi or vegetations can appear similar to a PFE. In these cases, a thorough clinical history (ie, history of myocardial infarction, persistent fever), clinical examination, laboratory tests (ie, persistence of infect parameters), and other image modalities (ie, CMR) can help establishing a definite diagnosis.^21^

Surgical excision of the tumor and its base is the treatment of choice in these patients. A recent report identified a possible recurrence of this type of tumor, thus making the clinical and imaging follow‐up of these patients necessary.^4^

## 4 CONCLUSION

Herein, we present a papillary fibroelastoma with an atypical localization, namely attached to the left ventricular outflow tract in a patient with a postexercise syncope. The coronary computed tomography completed the perioperative assessment of this patient providing an excellent description of the tumor and of the coronary circulation, thus avoiding an invasive diagnostic procedure prior to surgery.

## CONFLICT OF INTEREST

None declared.

## AUTHOR CONTRIBUTION

SG and GK: evaluated the patient and drafted the manuscript. SB: performed the coronary computed tomography and offered valuable intellectual input. FL: performed the histopathological analysis and offered valuable intellectual input. MK: performed the cardiac surgery and offered valuable intellectual input.

## ETHICAL STATEMENT

The patient provided written informed consent for the publication of her medical information in an anonymized form.

## References

[1] Giuşcă S , Jurcuţ R , Serban M , Popescu BA , Apetrei E , Ginghină C . Cardiac tumors: the experience of a tertiary cardiology center. Rom J Intern Med. 2007;45(4):333‐339. 18767408

[2] Bruce CJ . Cardiac tumours: diagnosis and management. Heart. 2011;97(2):151‐160. 21163893 10.1136/hrt.2009.186320

[3] Burke AP , Virmani R . Cardiac myxoma. A clinicopathologic study. Am J Clin Pathol. 1993;100(6):671‐680. 8249916 10.1093/ajcp/100.6.671

[4] Tamin SS , Maleszewski JJ , Scott CG , et al. Prognostic and bioepidemiologic implications of papillary fibroelastomas. J Am Coll Cardiol. 2015;65(22):2420‐2429. 26046736 10.1016/j.jacc.2015.03.569

[5] Salam KA , Rafeeque M , Hashim H , Mampilly N , Noone ML . Histology of thrombectomy specimen reveals cardiac tumor embolus in cryptogenic young stroke. J Stroke Cerebrovasc Dis. 2018;27(4):e70‐e72. 29246671 10.1016/j.jstrokecerebrovasdis.2017.11.015

[6] Sun JP , Asher CR , Yang XS , et al. Clinical and echocardiographic characteristics of papillary fibroelastomas: a retrospective and prospective study in 162 patients. Circulation. 2001;103(22):2687‐2693. 11390338 10.1161/01.cir.103.22.2687

[7] Hoey E , Mankad K , Puppala S , Gopalan D , Sivananthan MU . MRI and CT appearances of cardiac tumours in adults. Clin Radiol. 2009;64(12):1214‐1230. 19913133 10.1016/j.crad.2009.09.002

[8] Giusca S , Mereles D , Ochs A , et al. Incremental value of cardiac magnetic resonance for the evaluation of cardiac tumors in adults: experience of a high volume tertiary cardiology centre. Int J Cardiovasc Imaging. 2017;33(6):879‐888. 28138817 10.1007/s10554-017-1065-7

[9] Brignole M , Moya A , de Lange FJ , et al. 2018 ESC Guidelines for the diagnosis and management of syncope. Eur Heart J. 2018;39(21):1883‐1948. 29562304 10.1093/eurheartj/ehy037

[10] Colin GC , Gerber BL , Amzulescu M , Bogaert J . Cardiac myxoma: a contemporary multimodality imaging review. Int J Cardiovasc Imaging. 2018;34(11):1789‐1808. 29974293 10.1007/s10554-018-1396-z

[11] Silverman NA . Primary cardiac tumors. Ann Surg. 1980;191(2):127‐138. 7362282 10.1097/00000658-198002000-00001 PMC1345598

[12] Gowda RM , Khan IA , Nair CK , Mehta NJ , Vasavada BC , Sacchi TJ . Cardiac papillary fibroelastoma: a comprehensive analysis of 725 cases. Am Heart J. 2003;146(3):404‐410. 12947356 10.1016/S0002-8703(03)00249-7

[13] Grinda J‐M , Couetil JP , Chauvaud S , et al. Cardiac valve papillary fibroelastoma: surgical excision for revealed or potential embolization. J Thorac Cardiovasc Surg. 1999;117(1):106‐110. 9869763 10.1016/s0022-5223(99)70474-5

[14] Mutlu B , Eroğlu E , Bayrak F , Ipek G , Başaran Y . Papillary fibroelastoma of the aortic valve in a patient with syncope. Anadolu Kardiyol Derg. 2004;4(1):103‐104. 15033633

[15] Thongcharoen P , Laksanabunsong P , Thongtang V . Left ventricular outflow tract obstruction due to a left ventricular myxoma: a case report and review of the literature. J Med Assoc Thai. 1997;80(12):799‐806. 9470335

[16] Pazos‐López P , Pozo E , Siqueira ME , et al. Value of CMR for the differential diagnosis of cardiac masses. JACC Cardiovasc Imaging. 2014;7(9):896‐905. 25129516 10.1016/j.jcmg.2014.05.009

[17] Kim EY , Choe YH , Sung K , Park SW , Kim JH , Ko Y‐H . Multidetector CT and MR imaging of cardiac tumors. Korean J Radiol. 2009;10(2):164‐175. 19270863 10.3348/kjr.2009.10.2.164 PMC2651440

[18] Pindyck F , Peirce EC , Baron MG , Lukban SB . Embolization of left atrial myxoma after transseptal cardiac catheterization. Am J Cardiol. 1972;30(5):569‐571. 5073672 10.1016/0002-9149(72)90052-5

[19] Daveron E , Jain N , Kelley GP , et al. Papillary fibroelastoma and Lambl's excrescences: echocardiographic diagnosis and differential diagnosis. Echocardiogr Mt Kisco N. 2005;22(5):461‐463. 10.1111/j.1540-8175.2005.40063.x 15901304

[20] Kelle S , Chiribiri A , Meyer R , Fleck E , Nagel E . Images in cardiovascular medicine. Papillary fibroelastoma of the tricuspid valve seen on magnetic resonance imaging. Circulation. 2008;117(11):e190‐191. 18347215 10.1161/CIRCULATIONAHA.107.729731

[21] Prifti E , Ikonomi M , Veshti A , Demiraj A , Xhaxho R . Papillary fibroelastoma of the anterior leaflet of the mitral valve mimicking vegetation. Int J Surg Case Rep. 2015;10(14):19‐22. 10.1016/j.ijscr.2015.07.003 PMC4573212 26209756

